# With a little help from ferredoxin-NADP^+^ reductase: Enhancing photosynthetic cyclic electron transfer around PSI

**DOI:** 10.1093/plcell/koaf045

**Published:** 2025-03-07

**Authors:** Guy Levin

**Affiliations:** Assistant Features Editor, The Plant Cell, American Society of Plant Biologists; Department of Plant and Microbial Biology, University of California, Berkeley, CA 94720-3102, USA

In photosynthesis, light is harvested by chlorophyll and powers water oxidation by PSII. PSII then reduces thylakoid membrane-embedded plastoquinone (PQ) to form PQH_2_ using protons from the stromal side of the thylakoid membrane. PQH_2_ is oxidized by cytochrome *b*_6_*f* (cyt *b*_6_*f*) on the luminal side, releasing the protons into the lumen. ATP synthase utilizes the generated proton motive force (pmf) across the membrane to produce ATP (Figure). Cyt *b*_6_*f* then reduces plastocyanin, and light harvested by chlorophylls in PSI powers electron transfer from plastocyanin to ferredoxin (Fd) via PSI. In linear electron transfer (LET), Fd is oxidized by Fd-NADP^+^ reductase (FNR) to form NADPH, which, together with ATP, powers carbon fixation (Figure). In cyclic electron transfer (CET), electrons are shuttled back from PSI via Fd and NADPH to reduce PQ (Nawrocki et al. 2019). Thus, CET promotes the formation of pmf and ATP but not NADPH (Figure). CET provides additional energy required for carbon assimilation and enables a flexible ATP/NADPH balance following the organism's metabolic needs (Peltier et al. 2024). Despite its importance, many questions regarding the dynamic function of CEF remain open. For instance, to what extent is CEF required under different environmental conditions? Reports suggest that FNR location determines the LET/CET balance, promoting LET when soluble or bound to PSI or CET when bound to cyt *b6f* (Joliot and Johnson 2011). However, information on the interaction of FNR with cyt *b*_6_*f* is lacking. Moreover, because FNR is crucial for *C. reinhardtii* growth under both phototrophic and heterotrophic conditions, it is challenging to determine the importance of its interaction with cyt *b*_6_*f* as a CET facilitator.

**Figure.**
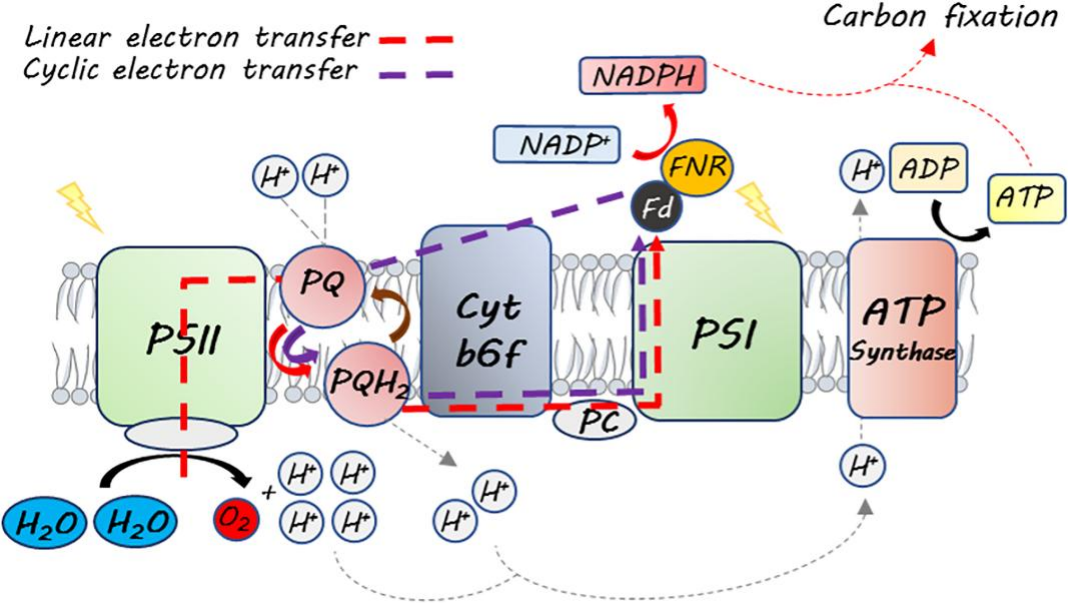
Photosynthetic linear and cyclic electron transfer pathways (LET and CET). PC, plastocyanin. Figure credit: G. Levin.

To determine the effect of FNR location on the balance between LET and CET, **Thomas Z. Emrich-Mills, Gustaf E. Degen, Matthew S. Proctor, and colleagues (Emrich-Mills et al. 2025)** use CRISPR-Cas9 to introduce FNR fused to the PSI subunit PSAF into the green algae Chlamydomonas (*C. reinhardtii*). The mutated cells showed impaired growth in phototrophic conditions but not when acetate was added as a carbon source. In agreement, PSII electron transfer rates were lower in the mutated cells under phototrophic conditions. These observations suggest that expressing PSAF-FNR negatively affects CO_2_ fixation by altering photosynthetic electron transfer. Chlorophyll fluorescence analysis of the mutated cells in oxic or anoxic conditions showed enhanced nonphotochemical quenching (NPQ) under low CO_2_ levels and compared with wild type, while the difference was smaller at higher CO_2_ concentrations. NPQ, which is induced by the acidification of the thylakoid lumen due to proton influx during photosynthesis, allows photosynthetic organisms to dissipate excess light safely as heat and protects the photosystems (Erickson et al. 2015). Indeed, electrochromatic shift measurements suggested a higher pmf in the mutated cells under high light (HL), driven by an increased proton flux. These results suggest that the mutated cells have a protective advantage over wild-type cells when growing under CET-inducing conditions, in this case, anoxia or low CO_2_. The enhanced pmf and NPQ could indicate increased CET activity, which transfers electrons back to the PQ pool and promotes proton translocation across the thylakoid membrane. Further electrochromatic shift and PSI activity analyses under anoxic conditions confirmed elevated CET in the mutated cells, suggesting that tethering FNR to PSI promotes CET rather than LET, as previously suggested.

CET provides photosynthetic organisms with additional ATP for CO_2_ fixation and acts to maintain the required ATP to NADPH ratio under given environmental conditions. Considering the rising CO_2_ levels in the atmosphere and the importance of CET in carbon assimilation, CET and other alternative photosynthetic electron transport pathways should be considered as potential targets for engineering plants with an enhanced capacity to capture CO_2_. Here, the authors provide an example of how gene editing techniques can be used to rewire electron transfer pathways in photosynthetic organisms. They demonstrate that, unexpectedly, CET can be enhanced at the expense of LET by tethering FNR to PSI, followed by a consequential rise in NPQ, which protects photosynthesis from HL and is also valuable from a biotechnological perspective (Leister 2023). This work provides important information for future research where CEF will be further fine-tuned in crops to provide optimal photosynthetic efficiency and maximal growth and CO_2_ sequestration rates.

## Recent related articles in *The Plant Cell*

- Croce et al. (2024) wrote a perspective that explores the latest advancements and approaches for improving photosynthesis in crops, aiming to enhance their yield.
- Eckardt et al. (2024) presented important open questions in photosynthesis research, including cyclic electron flow.
- Rolo et al. (2024) reviewed the latest knowledge about PSI assembly in vascular plants.

## Data Availability

No data analysis was performed during this work.

## Dive Curated Terms

The following phenotypic, genotypic, and functional terms are of significance to the work described in this paper:

ATP CHEBI: CHEBI:15422

NADPH CHEBI: CHEBI:16474

chlorophyll CHEBI: CHEBI:28966

PSAF Gramene: AT1G31330
PSAF Araport: AT1G31330
